# Massage and Medical Gymnastics

**Published:** 1921-01-08

**Authors:** 


### Massage and Medical Gymnastics.

IT is not very often that a society is in a position to dispense with annual subscriptions, but, from the opening of the New Year, this is the happy lot of the Chartered Society of Massage and Medical Gymnastics. All members and certificate-holders of the now superseded Incorporated Society of Trained Masseuses are entitled to become life members of the Chartered Society on payment of a registration fee of £2 2s., with half a guinea extra for such as were merely certificate-holders, and after this initial payment they are admitted for life to all the benefits of the Society without further payments. For this they may use the distinctive letters C.S.M.M.G.—likely to prove increasingly necessary as a passport to good practice—and wear the Society badge; may use the Society's registry for work; may attend lectures, demonstrations, and conferences arranged by the Chartered Society; may use the club-room at the headquarters, 157 Great Portland Street, W. 1, and benefit through the Members' Fund.

We are glad to hear that over 2,000 members and certificate-holders of the old I.S.T.M. have availed themselves of the privileges thus extended to them on such generous terms. The future of the Society now lies in the hands of its members. It is on them that the onus must rest of so combining and organising that club-houses may be formed in London and other cities, libraries built up, employment registries set afoot, and a corporate life be quickened. The trained nurse-masseuse finds herself working under widely different conditions to those she has been accustomed to when she takes up the work of massage, medical electricity, or medical gymnastics. Professional stimulus, professional co-operation are of the greatest possible service to her. Unless she keep up with the times she may find herself, within a few years of passing out of a first-rate training-centre, a mere laggard in the race.

Massage, with its kindred arts, is intensely interesting, because it is a live and growing branch of knowledge. It is to the lasting honour of the promoters of the Chartered Society that they recognised this aspect of it many years ago, when the torch was burning but faintly in this country, and have lifted the training of masseurs and masseuses to its rightful high place as one of the great educative processes available for the relief of suffering and the treatment of disease.

